# Survival prediction in acute myeloid leukemia using gene expression profiling

**DOI:** 10.1186/s12911-022-01791-z

**Published:** 2022-03-03

**Authors:** Binbin Lai, Yanli Lai, Yanli Zhang, Miao Zhou, Guifang OuYang

**Affiliations:** Department of Hematology, Ningbo First Hospital, 59 Liuting Road, Ningbo, 315000 Zhejiang Province China

**Keywords:** Acute myeloid leukemia, Random forest, The risk score, Overall survival

## Abstract

**Background:**

Acute myeloid leukemia (AML) is a genetically heterogeneous blood disorder. AML patients are associated with a relatively poor overall survival. The objective of this study was to establish a machine learning model to accurately perform the prognosis prediction in AML patients.

**Methods:**

We first screened for prognosis-related genes using Kaplan–Meier survival analysis in The Cancer Genome Atlas dataset and validated the results in the Oregon Health & Science University dataset. With a random forest model, we built a prognostic risk score using patient’s age, *TP53* mutation, ELN classification and normalized 197 gene expression as predictor variable. Gene set enrichment analysis was implemented to determine the dysregulated gene sets between the high-risk and low-risk groups. Similarity Network Fusion (SNF)-based integrative clustering was performed to identify subgroups of AML patients with different clinical features.

**Results:**

The random forest model was deemed the best model (area under curve value, 0.75). The random forest-derived risk score exhibited significant association with shorter overall survival in AML patients. The gene sets of pantothenate and coa biosynthesis, glycerolipid metabolism, biosynthesis of unsaturated fatty acids were significantly enriched in phenotype high risk score. SNF-based integrative clustering indicated three distinct subsets of AML patients in the TCGA cohort. The cluster3 AML patients were characterized by older age, higher risk score, more frequent *TP53* mutations, higher cytogenetics risk, shorter overall survival.

**Conclusions:**

The random forest-based risk score offers an effective method to perform prognosis prediction for AML patients.

**Supplementary Information:**

The online version contains supplementary material available at 10.1186/s12911-022-01791-z.

## Background

Acute myeloid leukemia (AML) is a genetically heterogeneous blood disorder characterized by distinct cytogenetic alterations, dysregulated gene expression and bone marrow failure [1]. In recent years, the incidence of the disease has dramatically increased, with the number of newly diagnosed cases reaching 119.57 × 10^3^ in 2017 alone [2]. AML patients are usually associated with an unfavorable prognosis, with 2- and 5-year survival rates of 32% and 24%, respectively [3]. The prognosis of the disease is highly correlated with patient age; older AML patients are more likely to have a relatively poor overall survival (OS), and the majority of elderly patients (over 70%) die within 1 year of AML diagnosis [4, 5].

European Leukemia-Net (ELN) has been widely utilized for prognosis stratification based on specific cytogenetic alterations in clinical settings. AML patients are stratified into favorable, adverse and intermediate prognostic subgroups following the ELN recommendations [6]. Moreover, several recent studies illustrated the prognostic importance of somatic mutations in critical cancer genes, such as mixed-lineage leukemia-partial tandem duplication, internal tandem duplication in Fms-like tyrosine kinase 3-internal tandem duplication (FLT3-ITD), tumor protein p53 (*TP53*) and ASXL transcriptional regulator 1 (*ASXL1*) mutations, and isocitrate dehydrogenase 1 (*IDH1*) mutations [7]. Furthermore, gene expression signatures have been proposed to be effective prognostic biomarkers and have shown promising potential for clinical applications [8, 9]. However, accurate prediction of patient prognosis remains a challenging task in AML.

Previous studies have mostly used certain genomic biomarkers [7, 10] or have performed linear regression analysis of gene expression signatures for prognosis prediction [8, 9]; however, these methods might not scale well to high-dimensional data. Machine learning techniques are known to handle high-dimensional data and offer more flexible alternatives for prognostic prediction using high-dimensional and heterogeneous data [11]. Recently, Karami et al. utilized various machine learning models to assess the survival of AML patients and showed that the Gradient Boosted Tree (GBT) model has the best performance in predicting the survival rate of AML patients. However, the established model lacks independent validation [12]. Orgueira et al. created a new machine learning model of AML survival using gene expression data and showed that the classifier achieved reasonable accuracy in predicting the survival rates of AML patients [13]. However, the accuracy of the classifier needs to be improved. Moreover, the molecular mechanism by which the classifier is predictive of AML patient survival remains unclear.

The objective of this study was to use machine learning methods to establish a prognostic model to accurately predict the prognosis of AML patients regardless of clinical characteristics. To this aim, we utilized the expression and clinical data of The Cancer Genome Atlas (TCGA) dataset [14] and screened for prognosis-associated clinical features and genes. A machine learning model was established using OS as the response variable, and prognosis-associated clinical features and genes were selected as predictor variables. The effectiveness of the machine learning model was independently validated in another Oregon Health & Science University (OHSU) dataset [15]. Finally, we performed similarity network fusion-based integrative clustering analysis and defined three distinct subgroups of AML patients showing considerable differences in clinicopathological characteristics and overall survival.

## Methods and materials

### Data acquisition and processing

We acquired RNA-seq expression data of 20,531 genes and clinical characteristics of AML patients from the TCGA database (TCGA dataset, n = 171) [14]. We removed the genes with missing rates of more than 90%. We also downloaded the gene expression data of 18,366 genes and clinical characteristics from Tyner’s study (the Oregon Health & Science University [OHSU] dataset, n = 403) [15] to independently validate the prognostic values of gene expression. We used Fisher’s exact test to study the correlations between the categorical features and patient OS and Student’s t test to analyze the correlations between quantitative clinical factors and patient mortality.

### Identification of prognosis-associated genes

We used the z score formula z = (x − $\overline{x }$)/s to normalize gene expression in the TCGA and OHSU cohorts. In the formula, x, $\overline{x }$ and s represent the gene expression value and the mean and standard deviation of the gene expression value, respectively. We followed Sha’s method to investigate the associations between gene expression and overall survival [8]. The AML patients were grouped into two subgroups, namely, the "high expression" and "low expression" groups, according to the median gene expression. The survival difference was analyzed by Kaplan–Meier curves and log-rank methods between the two subgroups using the survival package [16, 17]. Genes with *P* values < 0.05 were further grouped into risk genes and protective genes based on their correlations with OS. There were 12 AML patients whose follow-up times were 0 in the TCGA cohort, which caused several KM curves shown in the figures to not start at 1.

### Establishment and validation of the machine learning model

With Kaplan–Meier survival analysis, we identified 197 prognosis-associated genes common to the TCGA and OHSU cohorts. In this study, we aimed to build a machine learning model for prognosis prediction and used the caret package [18] to train four machine learning models, including support vector machine, random forest, neural network and ADABOOST classifier, using age, ELN classification, *TP53* mutation and normalized 197 gene expression as predictor variables and OS as response variables in the TCGA dataset. Sensitivity, specificity and accuracy values were computed by the caret package for the four models separately in the TCGA dataset using fivefold cross-validation. The median area under the curve (AUC) value was used for performance comparison among the four machine learning models in the TCGA dataset. The risk scores were predicted by the random forest model in the OHSU cohort for independent validation. Receiver operating characteristic (ROC) curves were plotted using the R package pROC to investigate the prognostic value of the random forest-based risk score (hereafter referred to as the risk score) [19]. We followed previously published studies [8, 9, 20] and dichotomized the risk scores into high- and low-risk groups according to the median risk score and compared their survival differences. We also implemented multivariate Cox regression analysis to examine whether the risk score was independently predictive of OS regardless of clinical features. Finally, a linear regression model was utilized to analyze the correlations between the risk score and clinical characteristics.

### Similarity network fusion-based integrative clustering analysis

SNFtool is an R package for similarity network fusion (SNF) that takes multiple views of a network and merges them into a combined view [21]. There were two different data types used in the SNF clustering, the first of which was clinical factors, including age, ELN classification, and *TP53* mutation, and the second of which was normalized to 197 gene expression levels. To integrate the two data types together, SNF was applied to preprocessed data using the *SNFtool* package. We utilized Fisher’s exact test for count variables and Student’s t test for quantitative clinical factors to characterize the differences between subgroups of patients. Kaplan–Meier survival analysis was performed among the three subgroups of AML patients using the R package survival [16]. *P* < 0.05 was predefined as statistically significant.

### Gene set enrichment analysis

The AML patients were divided into high- and low-risk groups based on the median risk score. Gene set enrichment analysis (GSEA) [22] was implemented to analyze the dysregulated gene sets between the high- and low-risk groups with the default parameters.

## Results

### Identification and validation of survival-related clinical characteristics

We first used different statistical methods to identify survival-related clinical characteristics. Detailed results regarding the association between clinical information and OS of the TCGA dataset are presented in Table 1. Patient age, *TP53* mutation and ELN classification were shown to be negatively related to OS in the TCGA cohort (*P* < 0.05 for all cases, Table 1). Similar results were also observed in the OHSU cohort (*P* < 0.05 for all cases, Additional file 1: Table S1). Chemotherapy, targeted therapy and bone marrow transplant were demonstrated to be protective factors for OS in AML patients (*P* < 0.05 for all cases, Table 1).

**Table1:** Association between the clinical features and patients’ mortality in 171 AML patients of the TCGA dataset

Variables	Group	Alive	Dead	*P* value	Statistical method
Age		49.63	58.86	0.00	Student t test
PBMBC		44.25	40.28	0.48	Student t test
Gender	Female	21	50	0.86	Fisher’s exact test
	Male	27	59		
European Leukemia Net classification	Favorable	10	7	0.03	Fisher’s exact test
	Intermediate	30	72		
	Poor	8	28		
*TP53* mutation	Mutant	0	14	0.003	Fisher’s exact test
	Wild-type	59	100		
*ASXL1* mutation	Mutant	0	3	0.55	Fisher’s exact test
	Wild-type	59	111		
*RUNX1* mutation	Mutant	3	13	0.27	Fisher’s exact test
	Wild-type	56	101		
*IDH1* mutation	Mutant	7	9	0.26	Fisher’s exact test
	Wild-type	41	100		
*IDH2* mutation	Mutant	5	12	1	Fisher’s exact test
	Wild-type	43	97		
*DNMT3A* mutation	Mutant	10	33	0.25	Fisher’s exact test
	Wild-type	38	76		
*NP1* mutation	Mutant	15	33	1	Fisher’s exact test
	Wild-type	33	76		
*CEBPA* mutation	Mutant	4	9	1	Fisher’s exact test
	Wild-type	44	100		
*FLT3* mutation	Mutant	12	32	0.7	Fisher’s exact test
	Wild-type	36	77		
Neoadjuvant treatment	Yes	12	31	0.7	Fisher’s exact test
	No	36	78		

### The random forest-based risk score is a negative prognostic factor in AML

**Fig. 1:**
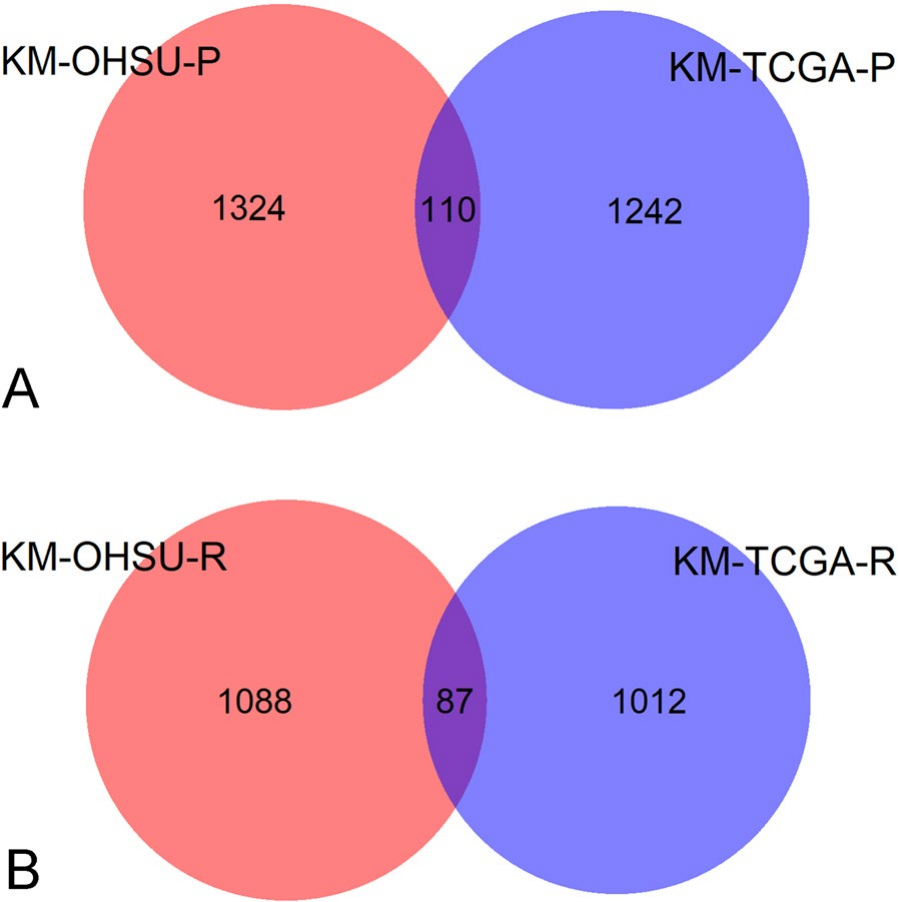
The overlap of survival-related genes between the OHSU and TCGA datasets. **A** The common protective genes determined by Kaplan–Meier survival analysis between the OHSU and TCGA datasets. **B** The common risk genes determined by Kaplan–Meier survival analysis between the OHSU and TCGA datasets

**Fig. 2:**
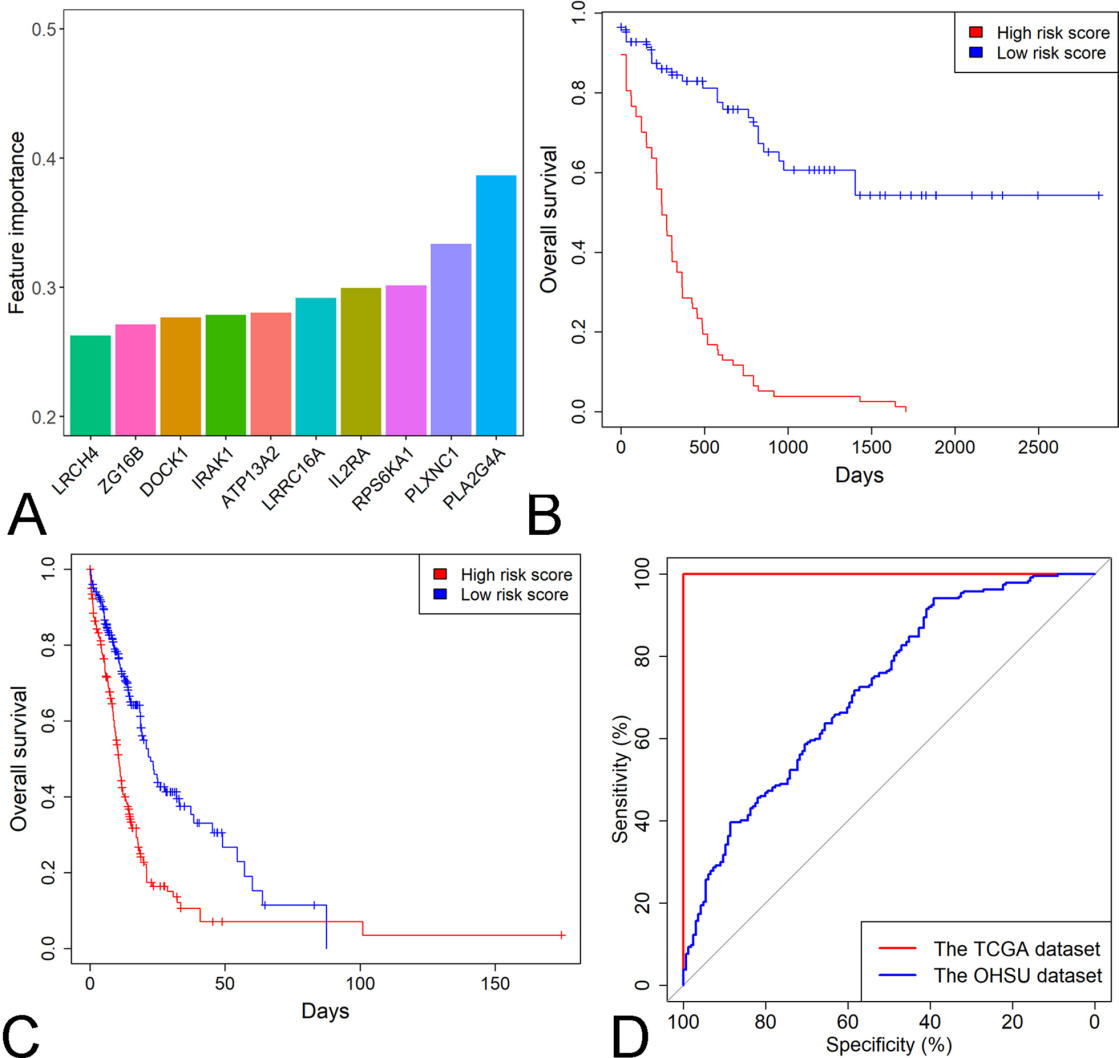
The risk score is a negative prognosticator in AML. **A** The top ten most important genes in the random forest model. **B** The difference in overall survival between the high- and low-risk groups stratified by the median risk score in the TCGA cohort. **C** The difference in overall survival between the high- and low-risk groups stratified by the median risk score in the OHSU cohort. **D** The ROC curves of the risk scores in the TCGA and OHSU datasets

Kaplan–Meier survival analysis identified 1352 protective genes and 1099 risk genes in the TCGA cohort (*P* < 0.05 for all cases, Fig. 1). The associations between the expression profiles of 2451 genes and OS were analyzed in the OHSU dataset. We confirmed that there were 110 protective genes and 87 risk genes in the OHSU cohort (*P* < 0.05 for all cases, Fig. 1). With the above 197 prognosis-associated genes, we trained four different machine learning models, including support vector machine, random forest, neural network and ADABOOST classifier, for the prediction of OS using age, ELN classification, *TP53* mutation and normalized expression of 197 genes as predictor variables and OS as the response variable in the TGCA dataset. We performed fivefold cross-validation to assess the performance of the four different machine learning models. Compared with the other three models, the random forest model showed the highest median AUC value (0.75) and was considered the optimal model for prognosis prediction (Additional file 2: Fig. S1). *PLA2G4A, PLXNC1, RPS6KA1, IL2RA, LRRC16A, ATP13A2, IRAK1, DOCK1, ZG16B* and *LRCH4* were the top ten most important features in the random forest model (Fig. 2A and Additional file 1: Table S2). Kaplan–Meier survival analysis showed that high risk scores were associated with worse OS in the TCGA cohort (*P* < 0.001, Fig. 2B). Then, we performed multivariate analysis between patient OS and survival-associated clinical features and the risk score and confirmed that a high risk score was a negative prognosticator in AML patients following adjustment for prognosis-associated clinical features (*P* < 0.001, odds ratio [OR]: 5.25, 95% confidence interval [CI]: 3.16–8.71, Table 2). The inverse association between the risk score and OS was verified in the OHSU dataset (Table 2 and Fig. 2C). The AUC values were 1 and 0.72 in the TCGA and OHSU cohorts, respectively (Fig. 2D). We also compared the performance of our random forest model with the 5-gene risk score in the OHSU dataset and demonstrated that our model outperformed the 5-gene risk score in the prediction of overall survival (AUC 0.72 vs. 0.65, Additional file 2: Fig. S2).

**Table2:** Multivariate analyses between OS and the risk score in the TCGA and OHSU datasets

	The TCGA dataset			The OHSU dataset			
Variable	OR	2.5%-97.5%CI	*P* value	Variable	OR	2.5–97.5%CI	*P* value
Age	1.02	1.01–1.04	0.004	Age	1.02	1.01–1.03	< 0.001
Cytogenetics risk	1.07	0.71–1.62	0.75	Cytogenetics risk	1.16	0.96–1.41	0.13
TP53.mutation	1.47	0.76–2.86	0.25	Chemotherapy	0.4	0.22–0.71	0.002
Risk score	5.25	3.16–8.71	< 0.001	Transplant	0.42	0.29–0.6	< 0.001
				Targeted therapy	0.91	0.66–1.26	0.58
				TP53.mutation	2.32	1.5–3.57	< 0.001
				Risk score	1.87	1.4–2.49	< 0.001

Notably, OR and CI refers to odds ratio and confidence interval respectively

### The risk score is an accurate prognostic predictor in AML

**Fig. 3:**
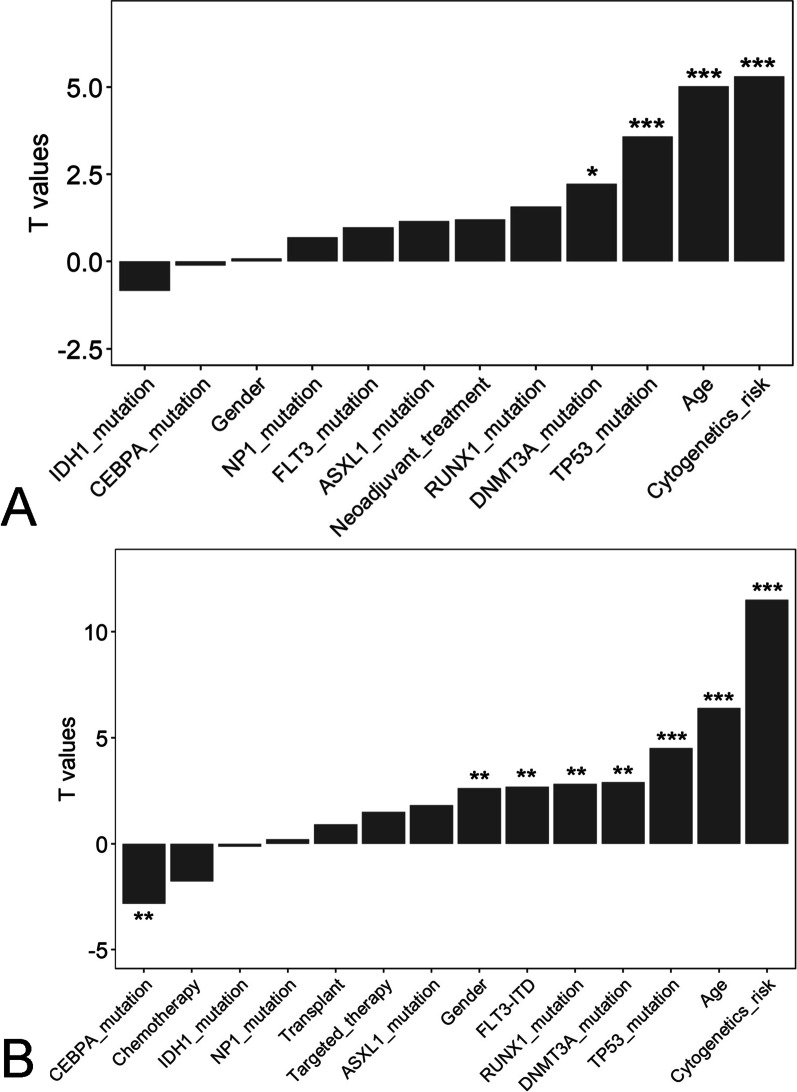
Linear regression model analysis of the correlations between risk score and clinical characteristics in the TCGA cohort (**A**) and the OHSU cohort (**B**). Notably, *, ** and *** refer to *P* values < 0.05, < 0.01 and 0.001, respectively

We carried out linear regression model analysis to characterize the association between clinical factors and the risk score. In the TCGA cohort, the risk score showed significantly positive correlations with patient age, ELN classification, and mutations in *DNMT3A* and *TP53* (*P* < 0.05 for all cases, Fig. 3A). Similar correlations were also observed in the OHSU cohort (*P* < 0.05 for all cases, Fig. 3B). Next, we aimed to analyze whether the negative correlation between the risk score and OS was independent of clinical characteristics. For each clinicopathological characteristic, we stratified LGG patients into two subgroups based on the median risk score and compared the OS difference using Kaplan–Meier survival analysis. A high risk score was significantly associated with shorter OS independent of age, sex, ELN classification, *DNMT3A* mutation, FLT3 mutation, *NP1* mutation, *IDH1* mutation, *CEBPA* mutation, and neoadjuvant treatment in the TCGA cohort (*P* < 0.05 for all cases, Additional file 2: Figs. S3–S6). As expected, similar results were confirmed in the OHSU cohort (*P* < 0.05 for all cases, Additional file 2: Figs. S7–S10), suggesting that the risk score could accurately predict prognosis regardless of clinicopathological characteristics.

### Gene set enrichment analysis

**Fig. 4:**
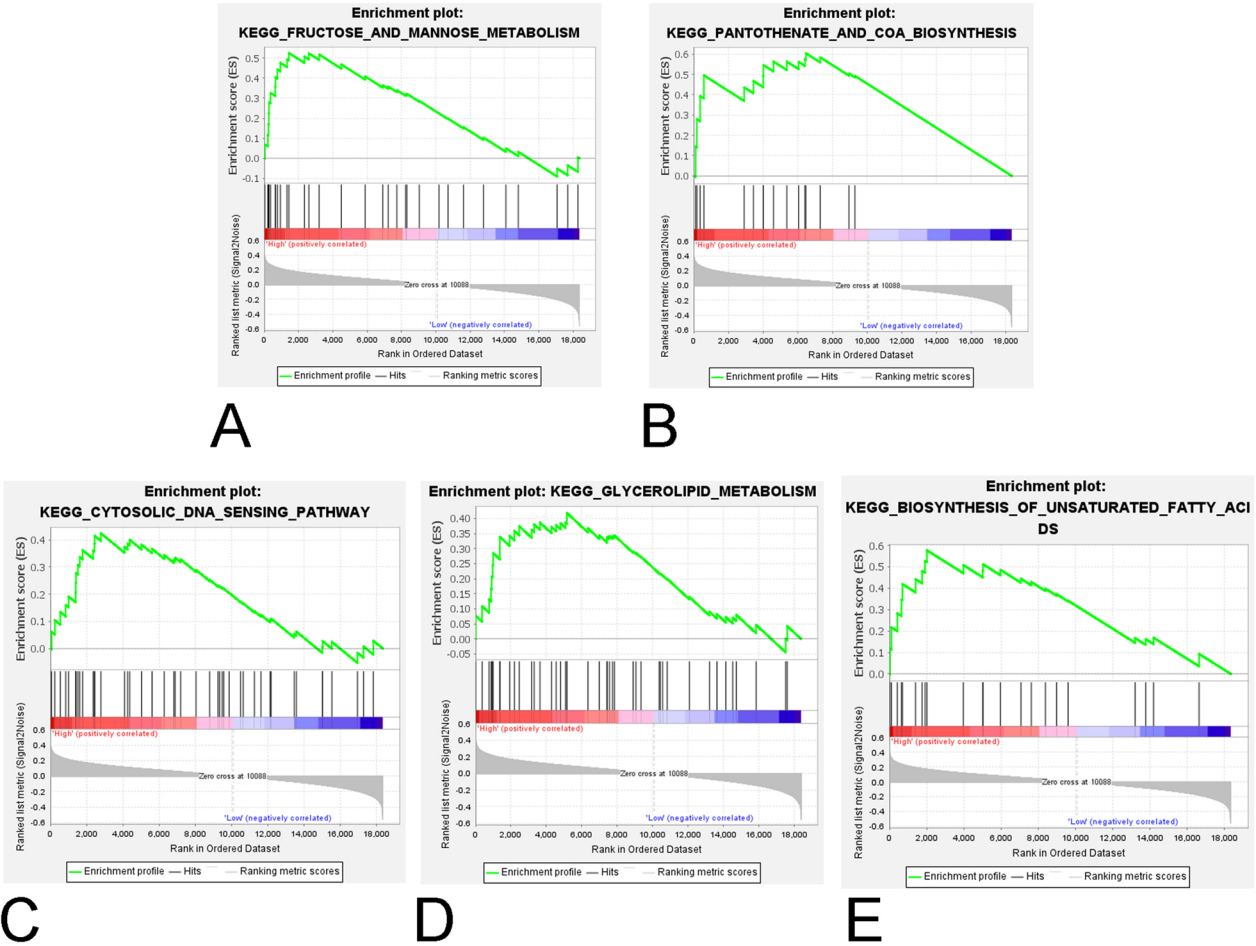
GSEA revealed significantly enriched pathways in the high risk score group, including fructose and mannose metabolism (**A**), pantothenate and CoA biosynthesis (**B**), cytosolic DNA sensing pathway (**C**), glycerolipid metabolism (**D**), and biosynthesis of unsaturated fatty acids (**E**). For each gene set, the positions of genes within the ranked list are shown as vertical bars along the x-axis of the GSEA plot. A negative enrichment score curve represents downregulated pathways, and a positive curve denotes upregulated pathways

The GSEA results showed that six signaling pathways were significantly enriched in the high risk score group, with fructose and mannose metabolism, pantothenate and CoA biosynthesis, cytosolic DNA sensing pathway, glycerolipid metabolism, and biosynthesis of unsaturated fatty acids being the top five most enriched pathways. Pathways such as glycosaminoglycan degradation and glycosylphosphatidylinositol gpi anchor biosynthesis were significantly enriched in the low risk score group (Fig. 4, *P* < 0.05 for all cases, Additional file 1: Table S3, the TCGA cohort). We also implemented GSEA in the OHSU cohort and determined that the gene sets of pantothenate and CoA biosynthesis, glycerolipid metabolism, and biosynthesis of unsaturated fatty acids were significantly enriched in the phenotype high risk score (*P* < 0.05 for all cases, Additional file 1: Table S4). These results suggest that the aforementioned pathways might largely contribute to the association between the risk score and OS.

### Similarity network fusion-based integrative clustering analysis

**Fig. 5:**
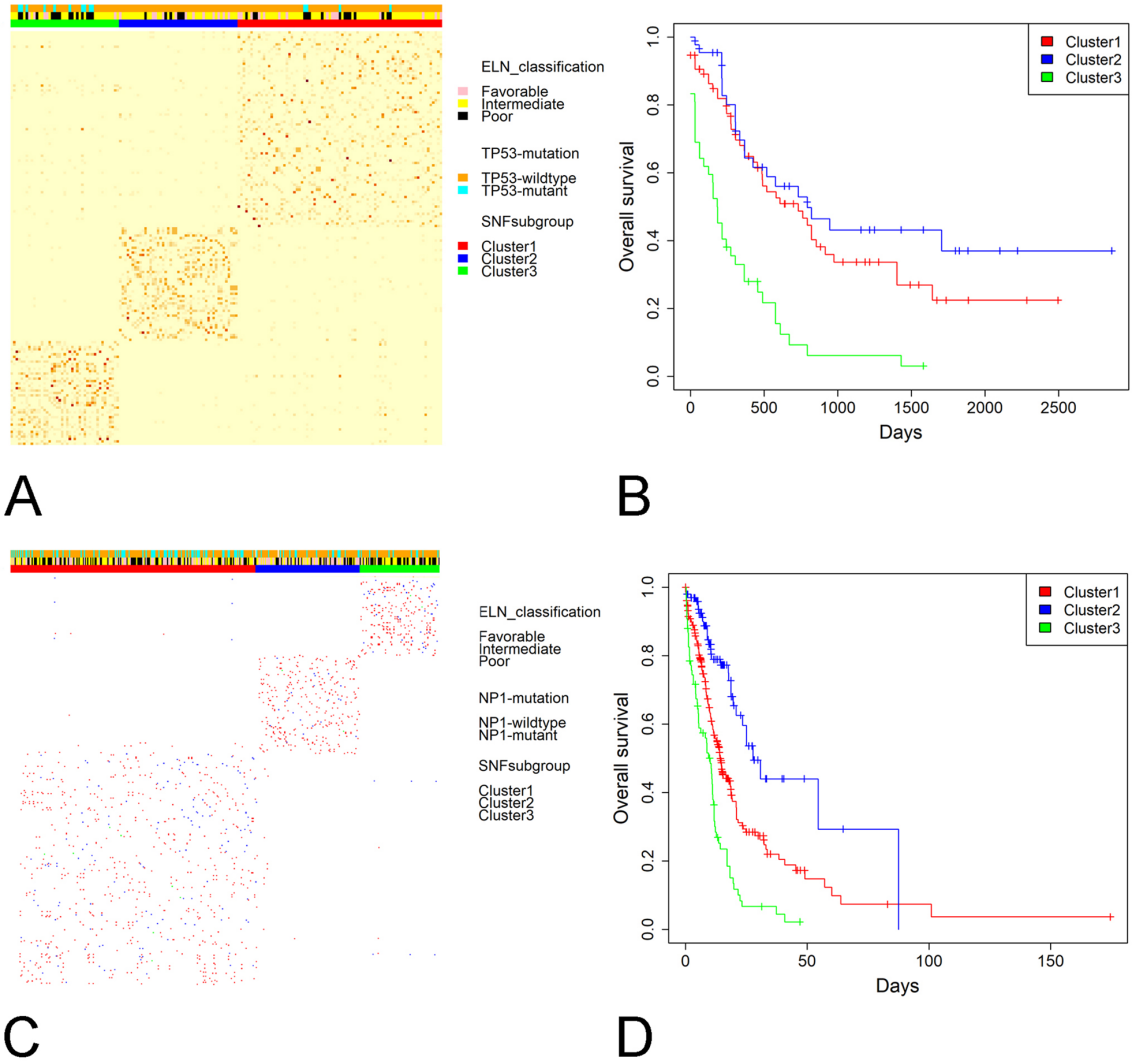
The SNF-based clustering analysis uncovered three classes of AML patients. **A** SNF-based clustering uncovered three classes of AML patients in the TCGA cohort. **B** The three subgroups of AML patients exhibited significant differences in overall survival in the TCGA cohort. **C** SNF-based clustering uncovered three classes of AML patients in the OHSU cohort. **D** The three subgroups of AML patients exhibited significant differences in overall survival in the OHSU cohort

The SNF clustering analysis indicated three classes of AML patients in the TCGA dataset (Fig. 5A). Compared to Cluster 1 and Cluster 2 patients, Cluster 3 AML patients were characterized by older age, higher risk score, more frequent *TP53* mutations, higher cytogenetics risk, and shorter OS (*P* values < 0.05 for all cases, Fig. 5B and Additional file 1: Table S5). We also conducted SNF clustering analysis in the OHSU dataset and uncovered three subgroups of AML patients (Fig. 5C). Similar to the results in the TCGA cohort, Cluster 3 samples exhibited older age, higher risk score, higher cytogenetics risk, more male cases, higher frequencies of *RUNX1* mutations, less frequent chemotherapy and bone marrow transplant, and shorter OS than Cluster 1 and Cluster 2 samples (*P* values < 0.05 for all cases, Fig. 5D and Additional file 1: Table S6).

## Discussion

The performance of the 2017 ELN classification to evaluate prognostic risk is well recognized in clinical settings [23]. Over the past five years, several gene expression signatures have been proposed to perform prognosis prediction and have shown potential clinical applicability in AML. For instance, Sha et al. selected five prognosis-associated genes, *CALCRL*, *PLA2G4A*, *FCHO2, DOCK1* and *LRCH4,* and used a linear regression model to combine the five genes and establish a 5-gene risk score [8]. In this study, we established a random forest-based risk score that accurately predicts prognosis regardless of clinicopathological characteristics in AML. Our method performed better than the 5-gene risk score (AUC, 0.65) in the OHSU cohort [24], suggesting that our method is superior to the existing risk stratification method. Given that the random forest-based risk score is independent of known prognosticators, such as ELN classification and *DNMT3A, FLT3, NP1, IDH1, and CEBPA* mutations, the risk score may be useful for the risk stratification of AML patients who have favorable or indeterminate cytogenetics risk or no mutations in key driver genes.

The mechanisms by which a high risk score is implicated in unfavorable prognosis in AML remain to be investigated. GSEA indicated that the gene sets of pantothenate and CoA biosynthesis, glycerolipid metabolism, and biosynthesis of unsaturated fatty acids were significantly enriched in the high risk score phenotype.

Glycerolipid metabolism and fatty acid metabolism play critical roles in the regulation of cell survival and proliferation [25, 26]. Several lipid signaling molecules and enhanced glycerolipid/free fatty acid cycling have been shown to enhance cell proliferation in many cell types [27–29]. We believe the prognostic importance of the risk score is, to a large extent, attributable to the upregulated expression of glycerolipid metabolism and the biosynthesis of unsaturated fatty acids in AML.

Compared to the two previously published machine learning methods [12, 13], our random forest model showed three main advantages. First, our random forest model was trained in the TCGA dataset and independently validated in the OHSU dataset, indicating a high reproducibility of survival prediction. Second, we demonstrated that the gene sets of pantothenate and CoA biosynthesis, glycerolipid metabolism, and biosynthesis of unsaturated fatty acids were significantly enriched in the high risk score phenotype, suggesting that these signaling pathways might partially contribute to the survival prediction. Third, we also performed SNF-based integrative clustering on AML patients and uncovered three distinct subsets of AML patients in the TCGA cohort. Cluster 3 AML patients were characterized by older age, higher risk score, more frequent *TP53* mutations, higher cytogenetics risk, and shorter overall survival. SNF-based integrative clustering might provide rational guidance for future treatment and follow-up for AML patients.

Among the 197 survival-related genes, many genes might have oncogenic functions in the tumorigenesis of cancers. For example, the overexpression of the *PLA2G4A* gene has been identified in several cancer types [30–33]. Silencing the expression of *PLA2G4A* considerably suppresses the survival and proliferation of lung cancer cells, glioblastoma cells [30], and colon cancer cells [33]. Dock family proteins, comprising 11 DOCK proteins (DOCK1-11), play crucial roles in the regulation of actin cytoskeleton, cell adhesion and migration [34]. Selective knockdown of *DOCK1* abolished cell motility and cell invasion and suppressed cancer growth and metastasis in a mouse model [35]. In line with the results in our study, higher *DOCK1* expression was a risk factor for overall survival in AML [36]. Last, knockdown of the two genes, *PLA2G4A* and *DOCK1,* caused significant reductions in cellular growth, invasion and tumorigenic capability; therefore, the two genes might become therapeutic targets for AML patients.

## Conclusion

Taken together, we developed a novel random forest-based risk score. The risk score outperforms established risk stratification method and is predictive of a poor OS in AML patients.

## Supplementary Information

**Additional file 1: Table S1.** Association between the clinical features and patients’ mortality in 403 AML patients of the OHSU dataset. **Table S2.** Feature importance in the random forest model. **Table S3.** The significantly up-regulated signalling pathways in the high or low risk score group of the TCGA cohort. **Table S4.** The significantly up-regulated signalling pathways in the high risk score group of the OHSU cohort. **Table S5.** The comparison of clinical characteristics among the three subgroups of AML patients in the TCGA dataset. **Table S6.** The comparison of clinical characteristics among the three subgroups of AML patients in the OHSU dataset.

**Additional file 2: Fig. S1.** The comparison of specificity, AUC and sensitivity values of the four machine learning models, including random forest (RF), support vector machine (SVM), ADABOOST classifier and neural network (NNET). **Fig. S2.** Comparison of performance of the random forest model and 5-gene risk score in the prediction of overall survival in the OHSU dataset. **Fig. S3.** Kaplan–Meier survival analysis of patients’ OS with the risk score in the subgroups of LGG patients stratified by the median patient age, gender and *CEBPA* mutation (**A**–**F**) of the TCGA cohort. **Fig. S4.** Kaplan–Meier survival analysis of patients’ OS with the risk score in the subgroups of LGG patients stratified by ELN classification, *TP53* and *IDH1* mutation (**A**–**F**) of the TCGA cohort. **Fig. S5.** Kaplan–Meier survival analysis of patients’ OS with the risk score in the subgroups of LGG patients stratified by *DNMT3A, FLT3* and *NP1* mutations (**A**–**F**) of the TCGA cohort. **Fig. S6.** Kaplan–Meier survival analysis of patients’ OS with the risk score in the subgroups of LGG patients stratified by bone marrow transplant and targeted therapy (A-F) of the TCGA cohort. **Fig. S7.** Kaplan–Meier survival analysis of patients’ OS with the risk score in the subgroups of LGG patients stratified by the median patient’s age, gender and *CEBPA* mutation (**A**–**F**) of the OHSU cohort. **Fig. S8.** Kaplan–Meier survival analysis of patients’ OS with the risk score in the subgroups of LGG patients stratified by ELN classification, *FLT3-ITD* and *NP1* mutation (**A**–**F**) of the OHSU cohort. **Fig. S9.** Kaplan–Meier survival analysis of patients’ RFS with the risk score in three subgroups of LGG patients stratified by *RUNX1*, *TP53* and *ASXL1* mutation (**A**–**F**) of the OHSU cohort. **Fig. S10.** Kaplan–Meier survival analysis of patients’ RFS with the risk score in three subgroups of LGG patients stratified by bone marrow transplant and targeted therapy (**A–D**) of the OHSU cohort.

## Data Availability

Gene expression data of 173 AML patients of the TCGA cohort and their clinical data were publicly available at https://figshare.com/s/7c683384c6e2add08262 (figshare ID: 13585235). The gene expression and clinical data of 405 AML patients the OHSU cohort used for the validation of survival analysis in our study were publicly available at https://figshare.com/s/7c683384c6e2add08262 (figshare ID: 13585235).

## Abbreviations

AML: Acute myeloid leukemia
TCGA: The Cancer Genome Altas
OHSU: The Oregon Health & Science University
ELN: European Leukemia-Net
OS: Overall survival
ROC: Receiver operating characteristic
AUC: Area under curve
LSC: Leukaemia stem cells
GSEA: Gene set enrichment analysis
OR: Odds ratio
CI: Confidence interval
