# Supplementary material for: Survival prediction in acute myeloid leukemia using gene expression profiling
Source: BMC Med Inform Decis Mak. 2022 Mar 3;22:57. doi: 10.1186/s12911-022-01791-z (PMC8892720; doi:10.1186/s12911-022-01791-z)
Supplement: Supplementary file 1 — Additional file 1: Table S1. Association between the clinical features and patients’ mortality in 403 AML patients of the OHSU dataset. Table S2. Feature importance in the random forest model. Table S3. The significantly up-regulated signalling pathways in the high or low risk score group of the TCGA cohort. Table S4. The significantly up-regulated signalling pathways in the high risk score group of the OHSU cohort. Table S5. The comparison of clinical characteristics among the three subgroups of AML patients in the TCGA dataset. Table S6. The comparison of clinical characteristics among the three subgroups of AML patients in the OHSU dataset. [file 12911_2022_1791_MOESM1_ESM.docx]

| Variables | Group | Alive | Dead | P value | Statistical method |
| --- | --- | --- | --- | --- | --- |
| Age |  | 49.18 | 61.82 | <0.001 | Student t test |
| PBMBC |  | 58.84 | 58.6 | 0.95 | Student t test |
| Gender | Female | 26 | 54 | 0.09 | Fisher’s exact test |
|  | Male | 77 | 94 |  |  |
| European Leukemia Net classification | Favourable | 69 | 40 | <0.001 | Fisher’s exact test |
|  | Intermediate | 48 | 94 |  |  |
|  | Poor | 42 | 103 |  |  |
| *TP53* mutation | Mutant | 3 | 29 | <0.001 | Fisher’s exact test |
|  | Wild-type | 164 | 209 |  |  |
| *ASXL1* mutation | Mutant | 11 | 20 | 0.57 | Fisher’s exact test |
|  | Wild-type | 156 | 218 |  |  |
| *RUNX1* mutation | Mutant | 15 | 31 | 0.27 | Fisher’s exact test |
|  | Wild-type | 152 | 207 |  |  |
| FLT3-IDT | Negative | 130 | 176 | 0.09 | Fisher’s exact test |
|  | Positive | 29 | 61 |  |  |
| CEBPA mutation | Negative | 61 | 82 | 0.83 | Fisher’s exact test |
|  | Positive | 12 | 14 |  |  |
| *IDH1* mutation | Negative | 61 | 88 | 0.19 | Fisher’s exact test |
|  | Positive | 14 | 11 |  |  |
| *DNMT3A* mutation | Negative | 35 | 53 | 0.61 | Fisher’s exact test |
|  | Positive | 25 | 31 |  |  |
| *NP1* mutation | Negative | 116 | 178 | 0.56 | Fisher’s exact test |
|  | Positive | 43 | 57 |  |  |
| Chemotherapy | Yes | 157 | 211 | 0.01 | Fisher’s exact test |
|  | No | 1 | 14 |  |  |
| Bone marrow transplant | Yes | 70 | 42 | <0.001 | Fisher’s exact test |
|  | No | 88 | 183 |  |  |
| Targeted therapy | Yes | 15 | 55 | <0.001 | Fisher’s exact test |
|  | No | 143 | 170 |  |  |

Additional file 1: Supplemental Table1. Association between the clinical features and patients’ mortality in 403 AML patients of the OHSU dataset

Supplementary table2. Feature importance in the random forest model

| Gene | | Feature importance |
| --- | --- | --- |
| PLA2G4A | 0.39 | |
| PLXNC1 | 0.33 | |
| RPS6KA1 | 0.30 | |
| IL2RA | 0.30 | |
| LRRC16A | 0.29 | |
| ATP13A2 | 0.28 | |
| IRAK1 | 0.28 | |
| DOCK1 | 0.28 | |
| ZG16B | 0.27 | |
| LRCH4 | 0.26 | |
| MTF2 | 0.26 | |
| CCND3 | 0.26 | |
| RECK | 0.25 | |
| COL19A1 | 0.25 | |
| CALCRL | 0.25 | |
| TGIF1 | 0.25 | |
| KIAA1549 | 0.25 | |
| GMPR2 | 0.25 | |
| HS3ST3B1 | 0.25 | |
| ACOT7 | 0.25 | |
| C14orf28 | 0.24 | |
| SYCE1 | 0.24 | |
| KLRG1 | 0.23 | |
| ASCC1 | 0.23 | |
| FNDC3A | 0.23 | |
| Age | 0.23 | |
| FYCO1 | 0.22 | |
| LSP1 | 0.22 | |
| PI4K2B | 0.22 | |
| PEF1 | 0.22 | |
| CD109 | 0.22 | |
| C14orf142 | 0.22 | |
| EFCAB4A | 0.22 | |
| ATP9B | 0.22 | |
| RHOBTB2 | 0.22 | |
| PEA15 | 0.22 | |
| ATP6V0E2 | 0.21 | |
| HSD17B11 | 0.21 | |
| RPL13AP6 | 0.21 | |
| EDAR | 0.21 | |
| G6PD | 0.21 | |
| PSMD4 | 0.21 | |
| PPP1R7 | 0.21 | |
| SCN5A | 0.20 | |
| SLC29A2 | 0.20 | |
| KCTD17 | 0.20 | |
| P2RX4 | 0.20 | |
| MED20 | 0.20 | |
| ZNF763 | 0.20 | |
| MED29 | 0.20 | |
| FCHO2 | 0.20 | |
| SCCPDH | 0.20 | |
| OTUD7B | 0.20 | |
| CCDC6 | 0.20 | |
| EIF3L | 0.20 | |
| NAB2 | 0.20 | |
| IGDCC4 | 0.20 | |
| MKRN1 | 0.19 | |
| HSD17B1 | 0.19 | |
| HS3ST3A1 | 0.19 | |
| RPL26 | 0.19 | |
| MDS2 | 0.19 | |
| ZNF277 | 0.19 | |
| MANBA | 0.19 | |
| RPS10P7 | 0.19 | |
| ZBTB4 | 0.19 | |
| CASP10 | 0.19 | |
| DHX32 | 0.19 | |
| ARMC8 | 0.19 | |
| WDR54 | 0.18 | |
| C21orf67 | 0.18 | |
| STAMBPL1 | 0.18 | |
| SYTL4 | 0.18 | |
| FADS1 | 0.18 | |
| ZNF700 | 0.18 | |
| CYTL1 | 0.18 | |
| CHMP4C | 0.18 | |
| LDLRAD3 | 0.18 | |
| BBS9 | 0.18 | |
| COX5B | 0.18 | |
| FAM46A | 0.18 | |
| CLDN19 | 0.18 | |
| SRC | 0.18 | |
| RNF8 | 0.18 | |
| C10orf128 | 0.18 | |
| ST13 | 0.18 | |
| CTAGE5 | 0.18 | |
| TPD52 | 0.18 | |
| COL2A1 | 0.18 | |
| SCHIP1 | 0.18 | |
| MPZL3 | 0.18 | |
| TSLP | 0.17 | |
| CASP2 | 0.17 | |
| TAS2R13 | 0.17 | |
| AKNAD1 | 0.17 | |
| FAM179A | 0.17 | |
| HAS3 | 0.17 | |
| GRID1 | 0.17 | |
| POLR3G | 0.17 | |
| ZNF708 | 0.17 | |
| DYM | 0.17 | |
| MXRA5 | 0.17 | |
| TADA3 | 0.17 | |
| UBE2G1 | 0.17 | |
| NRBP2 | 0.17 | |
| ORAI2 | 0.17 | |
| DUSP7 | 0.17 | |
| GNAZ | 0.17 | |
| VPRBP | 0.17 | |
| GLIS3 | 0.17 | |
| ADCY2 | 0.16 | |
| VNN1 | 0.16 | |
| RPSA | 0.16 | |
| TRNAU1AP | 0.16 | |
| EFCAB10 | 0.16 | |
| CNOT8 | 0.16 | |
| PODXL2 | 0.16 | |
| RBM44 | 0.16 | |
| RREB1 | 0.16 | |
| UBXN2B | 0.16 | |
| COL14A1 | 0.16 | |
| PPP1R9A | 0.16 | |
| ZNF254 | 0.16 | |
| CCNH | 0.15 | |
| MPV17L | 0.15 | |
| TCF15 | 0.15 | |
| EIF3E | 0.15 | |
| GALNT12 | 0.15 | |
| TCTA | 0.15 | |
| NAP1L1 | 0.15 | |
| FERMT3 | 0.15 | |
| OPTN | 0.15 | |
| CA13 | 0.15 | |
| KIAA0087 | 0.15 | |
| RNASEH2B | 0.15 | |
| SPPL3 | 0.15 | |
| CLEC4D | 0.15 | |
| FLOT1 | 0.15 | |
| BMI1 | 0.15 | |
| DAP3 | 0.15 | |
| MGP | 0.15 | |
| MAP4K4 | 0.15 | |
| DLX1 | 0.15 | |
| FRMD6 | 0.14 | |
| CDH11 | 0.14 | |
| IGF2 | 0.14 | |
| ACADSB | 0.14 | |
| DENND2C | 0.14 | |
| TTC38 | 0.14 | |
| PSMB8 | 0.14 | |
| FAM189B | 0.14 | |
| SNAP47 | 0.14 | |
| FBXL17 | 0.14 | |
| MESDC2 | 0.14 | |
| LPAR4 | 0.14 | |
| SLC22A3 | 0.14 | |
| RMND5B | 0.14 | |
| CXCL12 | 0.14 | |
| HOXB9 | 0.14 | |
| ZNF107 | 0.14 | |
| S100A1 | 0.14 | |
| ZMAT3 | 0.14 | |
| IL33 | 0.14 | |
| HCP5 | 0.13 | |
| INF2 | 0.13 | |
| MTMR12 | 0.13 | |
| GOLGA3 | 0.13 | |
| CHD1 | 0.13 | |
| EIF4B | 0.13 | |
| CD160 | 0.13 | |
| JARID2 | 0.13 | |
| FMO2 | 0.12 | |
| GPR116 | 0.12 | |
| BEND4 | 0.12 | |
| WDSUB1 | 0.12 | |
| CHL1 | 0.12 | |
| PTP4A1 | 0.12 | |
| SPAG6 | 0.12 | |
| SCNN1B | 0.12 | |
| HNRNPA1L2 | 0.11 | |
| TMEM132C | 0.11 | |
| PACS2 | 0.11 | |
| BFSP1 | 0.11 | |
| LPAR3 | 0.10 | |
| PARVA | 0.10 | |
| FRZB | 0.10 | |
| NNMT | 0.10 | |
| COL3A1 | 0.10 | |
| HIST1H3G | 0.10 | |
| NPR1 | 0.10 | |
| NTRK2 | 0.09 | |
| UNCX | 0.09 | |
| NTN4 | 0.09 | |
| SOX9 | 0.08 | |
| SNAI2 | 0.08 | |
| Cytogenetics_risk | 0.08 | |
| ELTD1 | 0.07 | |
| CT45A5 | 0.06 | |
| SNORA45 | 0.06 | |
| TP53 mutation | 0.02 | |

Supplementary table3. The significantly up-regulated signalling pathways in the high or low risk score group of the TCGA cohort

| KEGG pathway name | Pathway size | Enrichement score | Normalized enrichment score | P value | Q value |
| --- | --- | --- | --- | --- | --- |
| *Gene sets enriched in phenotype high risk score* |  |  |  |  |  |
| FRUCTOSE_AND_MANNOSE_METABOLISM | 32 | 0.53 | 1.73 | 0.01 | 1.00 |
| PANTOTHENATE_AND_COA_BIOSYNTHESIS | 16 | 0.60 | 1.69 | 0.02 | 0.96 |
| CYTOSOLIC_DNA_SENSING_PATHWAY | 45 | 0.42 | 1.55 | 0.02 | 0.98 |
| GLYCEROLIPID_METABOLISM | 44 | 0.42 | 1.49 | 0.03 | 0.88 |
| BIOSYNTHESIS_OF_UNSATURATED_FATTY_ACIDS | 22 | 0.58 | 1.60 | 0.04 | 0.88 |
| STEROID_BIOSYNTHESIS | 16 | 0.63 | 1.67 | 0.04 | 0.74 |
| *Gene sets enriched in phenotype low risk score* |  |  |  |  |  |
| GLYCOSAMINOGLYCAN_DEGRADATION | 20 | -0.65 | -1.65 | 0.02 | 0.94 |
| GLYCOSYLPHOSPHATIDYLINOSITOL_GPI_ANCHOR_BIOSYNTHESIS | 25 | -0.52 | -1.58 | 0.04 | 0.76 |

Supplementary table4. The significantly up-regulated signalling pathways in the high risk score group of the OHSU cohort

| KEGG pathway name | Pathway size | Enrichement score | Normalized enrichment score | P value | Q value |
| --- | --- | --- | --- | --- | --- |
| GLYCEROLIPID_METABOLISM | 40 | 0.50 | 1.72 | 0.00 | 0.51 |
| PANTOTHENATE_AND_COA_BIOSYNTHESIS | 15 | 0.65 | 1.68 | 0.01 | 0.41 |
| GALACTOSE_METABOLISM | 22 | 0.63 | 1.74 | 0.02 | 1.00 |
| FATTY_ACID_METABOLISM | 33 | 0.54 | 1.72 | 0.02 | 0.72 |
| VALINE_LEUCINE_AND_ISOLEUCINE_DEGRADATION | 43 | 0.56 | 1.71 | 0.02 | 0.39 |
| BIOSYNTHESIS_OF_UNSATURATED_FATTY_ACIDS | 20 | 0.65 | 1.67 | 0.02 | 0.36 |
| GLYCOLYSIS_GLUCONEOGENESIS | 44 | 0.50 | 1.64 | 0.03 | 0.32 |
| PEROXISOME | 72 | 0.42 | 1.55 | 0.04 | 0.45 |
| PROPANOATE_METABOLISM | 31 | 0.55 | 1.57 | 0.04 | 0.43 |
| BASE_EXCISION_REPAIR | 34 | 0.54 | 1.65 | 0.04 | 0.35 |

Supplementary table5. The comparison of clinical characteristics among the three subgroups of AML patients in the TCGA dataset

| Variables | Group | Cluster1 | Cluster2 | Cluster3 | Cluster1 VS Cluster2 | Cluster1 VS Cluster3 | | | Cluster2 VS Cluster3 | Between group comparison |
| --- | --- | --- | --- | --- | --- | --- | --- | --- | --- | --- |
| Age |  | 58.05 | 33.45 | 73.77 | P <0.001 for all cases | | | | | Student t test |
| Risk score |  | 0.64 | 0.56 | 0.65 | 0.19 | <0.001 | | | <0.001 | Student t test |
| Gender | Female | 39 | 21 | 18 | P > 0.05 for all cases | | | | | Fisher’s exact test |
|  | Male | 42 | 26 | 25 |  |  | | |  |  |
| Cytogenetic risk | Favorable | 15 | 12 | 5 | 0.54 | 0.14 | | | 0.02 | Fisher’s exact test |
|  | Intermediate | 51 | 29 | 23 |  |  | | |  |  |
|  | Poor | 15 | 6 | 15 |  |  | | |  |  |
| *IDH1* mutation | Wild-type | 72 | 41 | 42 | P > 0.05 for all cases | | | | | Fisher’s exact test |
|  | Mutant | 9 | 6 | 1 |  |  | | |  |  |
| *DNMT3A* mutation | Wild-type | 55 | 39 | 35 | P > 0.05 for all cases | | | | | Fisher’s exact test |
|  | Mutant | 26 | 8 | 8 |  |  | | |  |  |
| *NP1* mutation | Wild-type | 57 | 35 | 33 | P > 0.05 for all cases | | | | | Fisher’s exact test |
|  | Mutant | 24 | 12 | 10 |  |  | | |  |  |
| *CEBPA* mutation | Wild-type | 76 | 40 | 42 | P > 0.05 for all cases | | | | | Fisher’s exact test |
|  | Mutant | 5 | 7 | 1 |  |  | | |  |  |
| *FLT3* mutation | Wild-type | 55 | 33 | 35 | P > 0.05 for all cases | | | | | Fisher’s exact test |
|  | Mutant | 26 | 14 | 8 |  |  | | |  |  |
| TP53 mutation | Wild-type | 77 | 47 | 33 | 0.3 | | 0.005 | <0.001 | | Fisher’s exact test |
|  | Mutant | 4 | 0 | 10 |  |  | | |  |  |
| ASXL1 mutation | Wild-type | 80 | 47 | 41 | P > 0.05 for all cases | | | | | Fisher’s exact test |
|  | Mutant | 1 | 0 | 2 |  |  | | |  |  |
| RUNX1 mutation | Wild-type | 75 | 44 | 36 | P > 0.05 for all cases | | | | | Fisher’s exact test |
|  | Mutant | 6 | 3 | 7 |  |  | | |  |  |
| Neoadjuvant treatment | Yes | 59 | 33 | 35 | P > 0.05 for all cases | | | | | Fisher’s exact test |
|  | No | 22 | 14 | 8 |  |  | | |  |  |

| Variables | Group | Cluster1 | Cluster2 | Cluster3 | Cluster1 VS Cluster2 | | Cluster1 VS Cluster3 | | Cluster2 VS Cluster3 | Between group comparison |
| --- | --- | --- | --- | --- | --- | --- | --- | --- | --- | --- |
| Age |  | 60.48 | 31.45 | 77.45 | P < 0.05 for all cases | | | | | Student t test |
| Risk score |  | 0.64 | 0.56 | 0.65 | <0.001 | | 0.3 | | <0.001 | Student t test |
| Gender | Female | 103 | 54 | 19 | 0.09 | | 0.003 | | <0.001 | Fisher’s exact test |
|  | Male | 127 | 44 | 56 |  | |  | |  |  |
| Cytogenetic risk | Favorable | 63 | 45 | 9 | P < 0.05 for all cases | | | | | Fisher’s exact test |
|  | Intermediate | 85 | 28 | 28 |  | |  | |  |  |
|  | Poor | 82 | 25 | 38 |  | |  | |  |  |
| *IDH1* mutation | Wild-type | 85 | 41 | 30 | P > 0.05 for all cases | | | | | Fisher’s exact test |
|  | Mutant | 18 | 3 | 4 |  | |  | |  |  |
| *DNMT3A* mutation | Wild-type | 43 | 31 | 21 | <0.001 | | 0.009 | | 0.51 | Fisher’s exact test |
|  | Mutant | 45 | 5 | 6 |  | |  | |  |  |
| *NP1* mutation | Wild-type | 159 | 77 | 65 | 0.04 | | 0.003 | | 0.31 | Fisher’s exact test |
|  | Mutant | 71 | 19 | 10 |  | |  | |  |  |
| *CEBPA* mutation | Wild-type | 86 | 40 | 24 | P > 0.05 for all cases | | | | | Fisher’s exact test |
|  | Mutant | 11 | 10 | 5 |  | |  | |  |  |
| *FLT3* mutation | Wild-type | 170 | 75 | 64 | 0.68 | | 0.04 | | 0.18 | Fisher’s exact test |
|  | Mutant | 60 | 23 | 11 |  | |  | |  |  |
| *TP53* | Wild-type | 1 | 24 | 7 | P > 0.05 for all cases | | | | | Fisher’s exact test |
|  | Mutant | 119 | 227 | 27 |  | |  | |  |  |
| *ASXL1* | Wild-type | 215 | 96 | 61 | 0.11 | 0.005 | | <0.001 | | Fisher’s exact test |
|  | Mutant | 15 | 2 | 14 |  | |  | |  |  |
| *RUNX1* | Wild-type | 204 | 93 | 60 | 0.1 | | 0.08 | | 0.003 | Fisher’s exact test |
|  | Mutant | 26 | 5 | 15 |  | |  | |  |  |
| Chemotherapy | Yes | 218 | 98 | 58 | 0.18 | 0.002 | | <0.001 | | Fisher’s exact test |
|  | No | 6 | 0 | 9 |  | |  | |  |  |
| Bone marrow transplant | Yes | 78 | 33 | 1 | 0.9 | | <0.001 | | <0.001 | Fisher’s exact test |
|  | No | 146 | 65 | 66 |  | |  | |  |  |
| Targeted therapy | Yes | 39 | 16 | 16 | P > 0.05 for all cases | | | | | Fisher’s exact test |
|  | No | 185 | 82 | 51 |  | |  | |  |  |

Supplementary Table6. The comparison of clinical characteristics among the three subgroups of AML patients in the OHSU dataset
