# Supplementary material for: Survival prediction in acute myeloid leukemia using gene expression profiling
Source: BMC Med Inform Decis Mak. 2022 Mar 3;22:57. doi: 10.1186/s12911-022-01791-z (PMC8892720; doi:10.1186/s12911-022-01791-z)
Supplement: Supplementary file 2 — Additional file 2: Fig. S1. The comparison of specificity, AUC and sensitivity values of the four machine learning models, including random forest (RF), support vector machine (SVM), ADABOOST classifier and neural network (NNET). Fig. S2. Comparison of performance of the random forest model and 5-gene risk score in the prediction of overall survival in the OHSU dataset. Fig. S3. Kaplan–Meier survival analysis of patients’ OS with the risk score in the subgroups of LGG patients stratified by the median patient age, gender and CEBPA mutation (A–F) of the TCGA cohort. Fig. S4. Kaplan–Meier survival analysis of patients’ OS with the risk score in the subgroups of LGG patients stratified by ELN classification, TP53 and IDH1 mutation (A–F) of the TCGA cohort. Fig. S5. Kaplan–Meier survival analysis of patients’ OS with the risk score in the subgroups of LGG patients stratified by DNMT3A, FLT3 and NP1 mutations (A–F) of the TCGA cohort. Fig. S6. Kaplan–Meier survival analysis of patients’ OS with the risk score in the subgroups of LGG patients stratified by bone marrow transplant and targeted therapy (A-F) of the TCGA cohort. Fig. S7. Kaplan–Meier survival analysis of patients’ OS with the risk score in the subgroups of LGG patients stratified by the median patient’s age, gender and CEBPA mutation (A–F) of the OHSU cohort. Fig. S8. Kaplan–Meier survival analysis of patients’ OS with the risk score in the subgroups of LGG patients stratified by ELN classification, FLT3-ITD and NP1 mutation (A–F) of the OHSU cohort. Fig. S9. Kaplan–Meier survival analysis of patients’ RFS with the risk score in three subgroups of LGG patients stratified by RUNX1, TP53 and ASXL1 mutation (A–F) of the OHSU cohort. Fig. S10. Kaplan–Meier survival analysis of patients’ RFS with the risk score in three subgroups of LGG patients stratified by bone marrow transplant and targeted therapy (A–D) of the OHSU cohort. [file 12911_2022_1791_MOESM2_ESM.docx]

**Additional file 2**


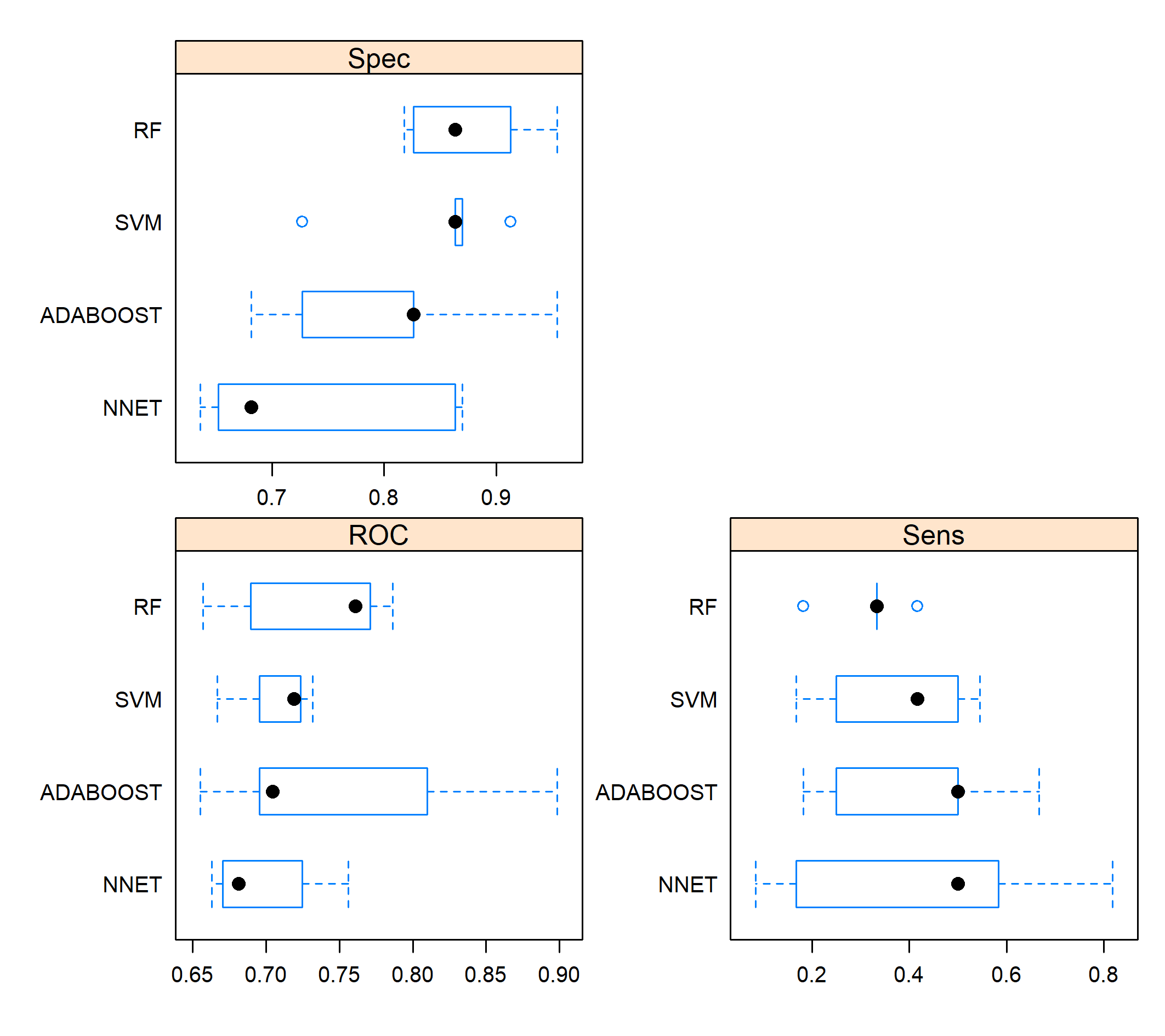
Supplementary Figure1. The comparison of specificity, AUC and sensitivity values of the four machine learning models, including random forest (RF), support vector machine(SVM), ADABOOST classifier and neural network (NNET).


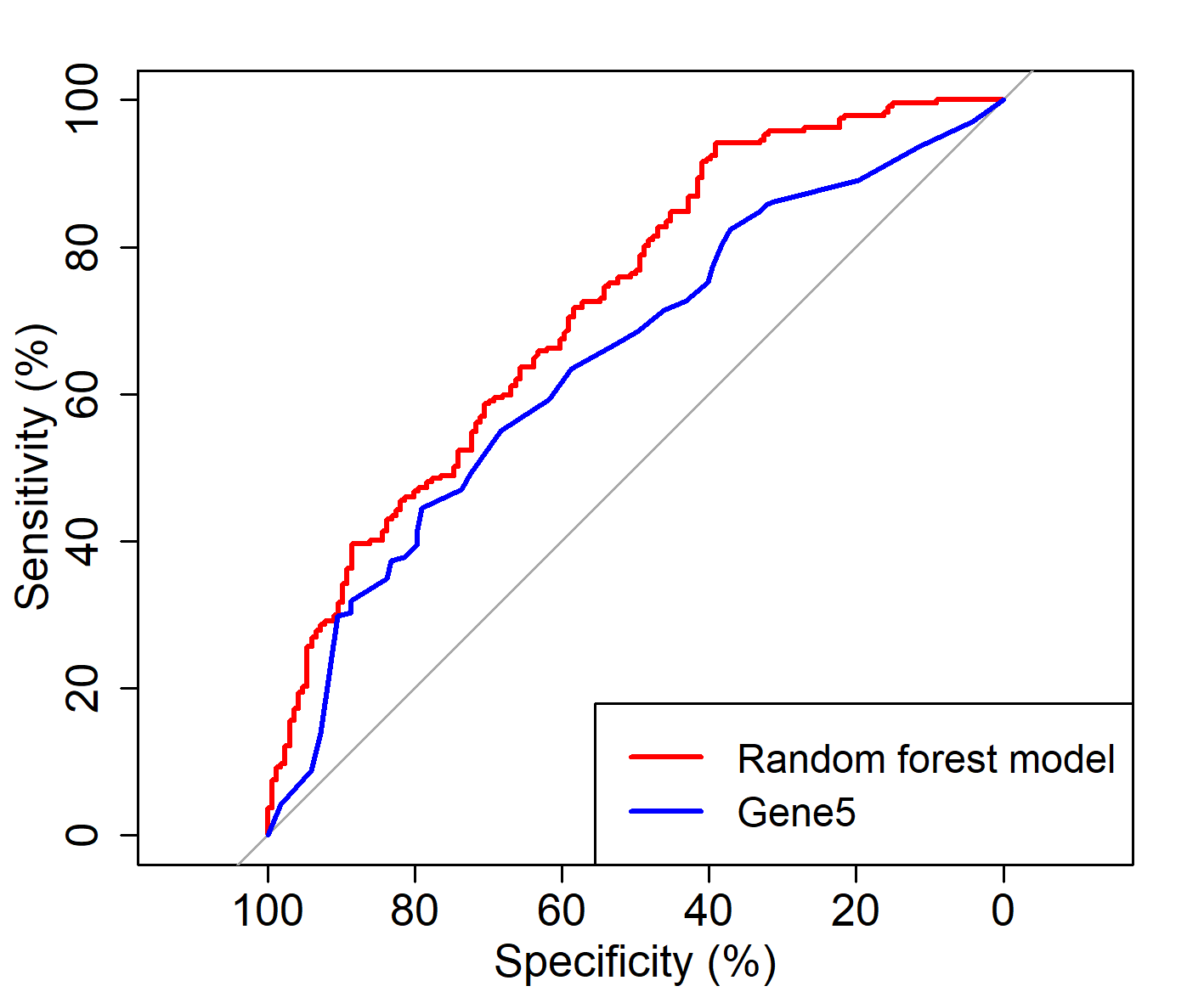


Supplementary Figure2. Comparison of performance of the random forest model and 5-gene risk score in the prediction of overall survival in the OHSU dataset.


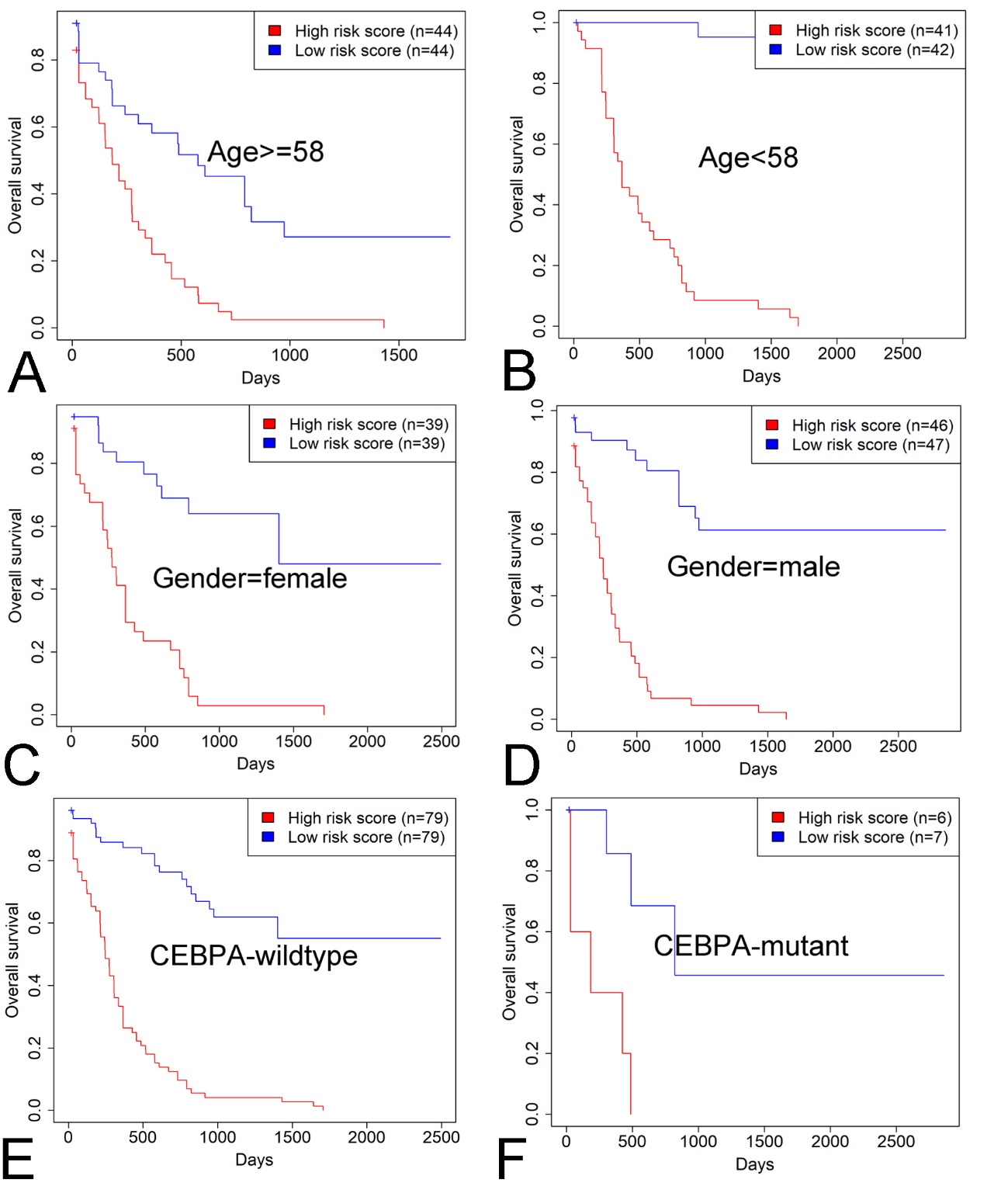


Supplementary Figure3. Kaplan-Meier survival analysis of patients’ OS with the risk score in the subgroups of LGG patients stratified by the median patient age, gender and *CEBPA* mutation (A-F) of the TCGA cohort.


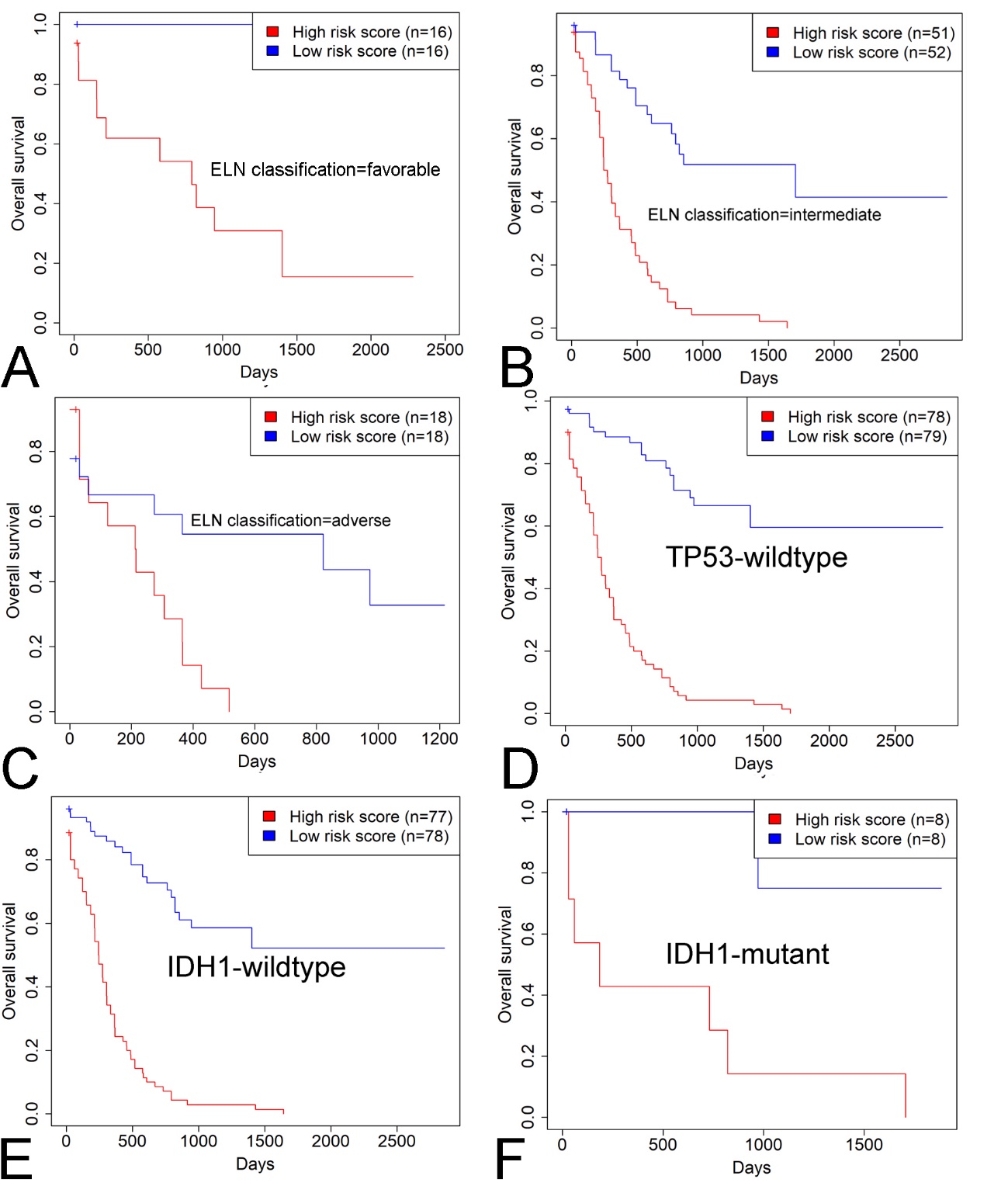
Supplementary Figure4. Kaplan-Meier survival analysis of patients’ OS with the risk score in the subgroups of LGG patients stratified by ELN classification, *TP53* and *IDH1* mutation (A-F) of the TCGA cohort.


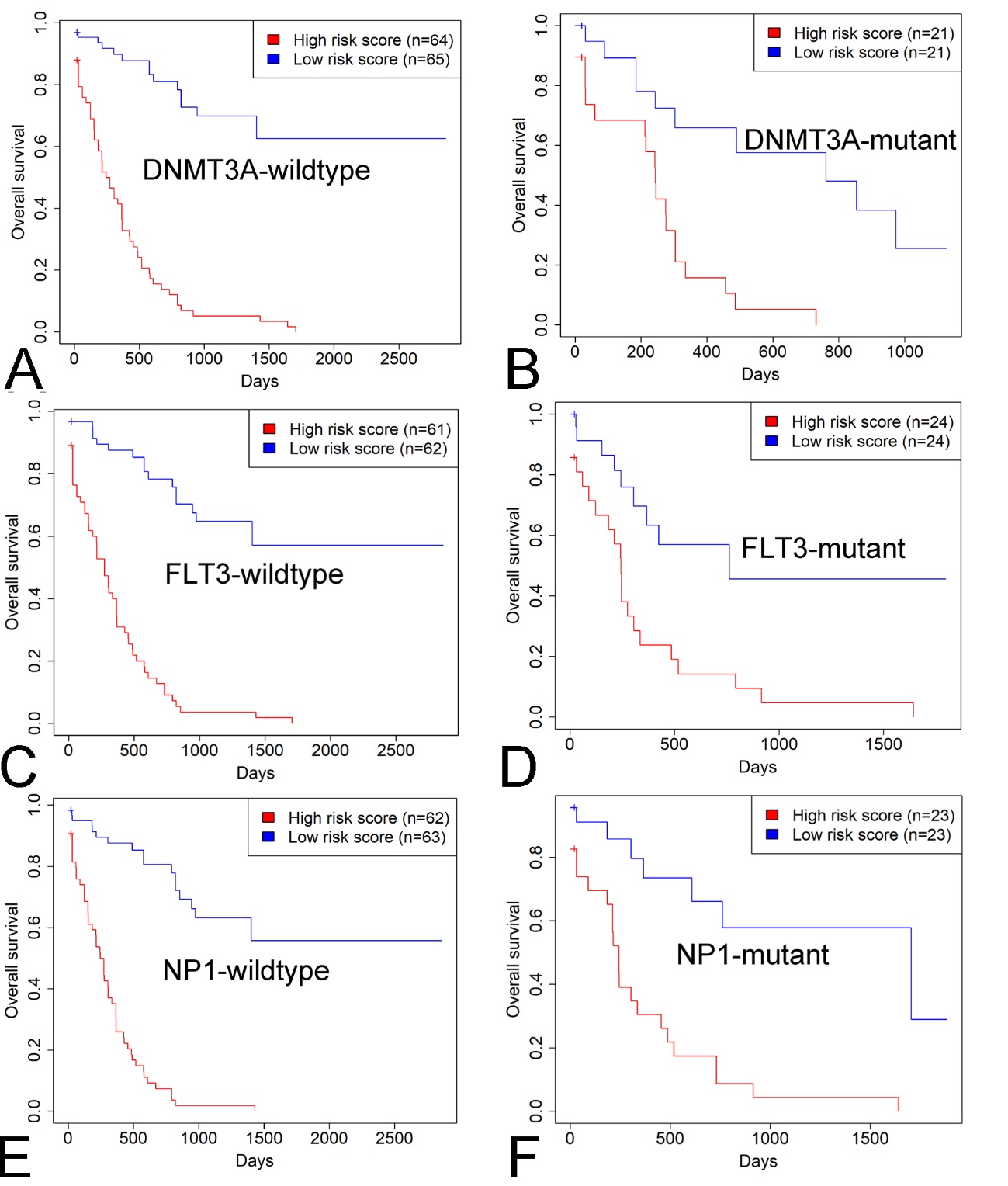
Supplementary Figure5. Kaplan-Meier survival analysis of patients’ OS with the risk score in the subgroups of LGG patients stratified by *DNMT3A, FLT3* and *NP1* mutations (A-F) of the TCGA cohort.


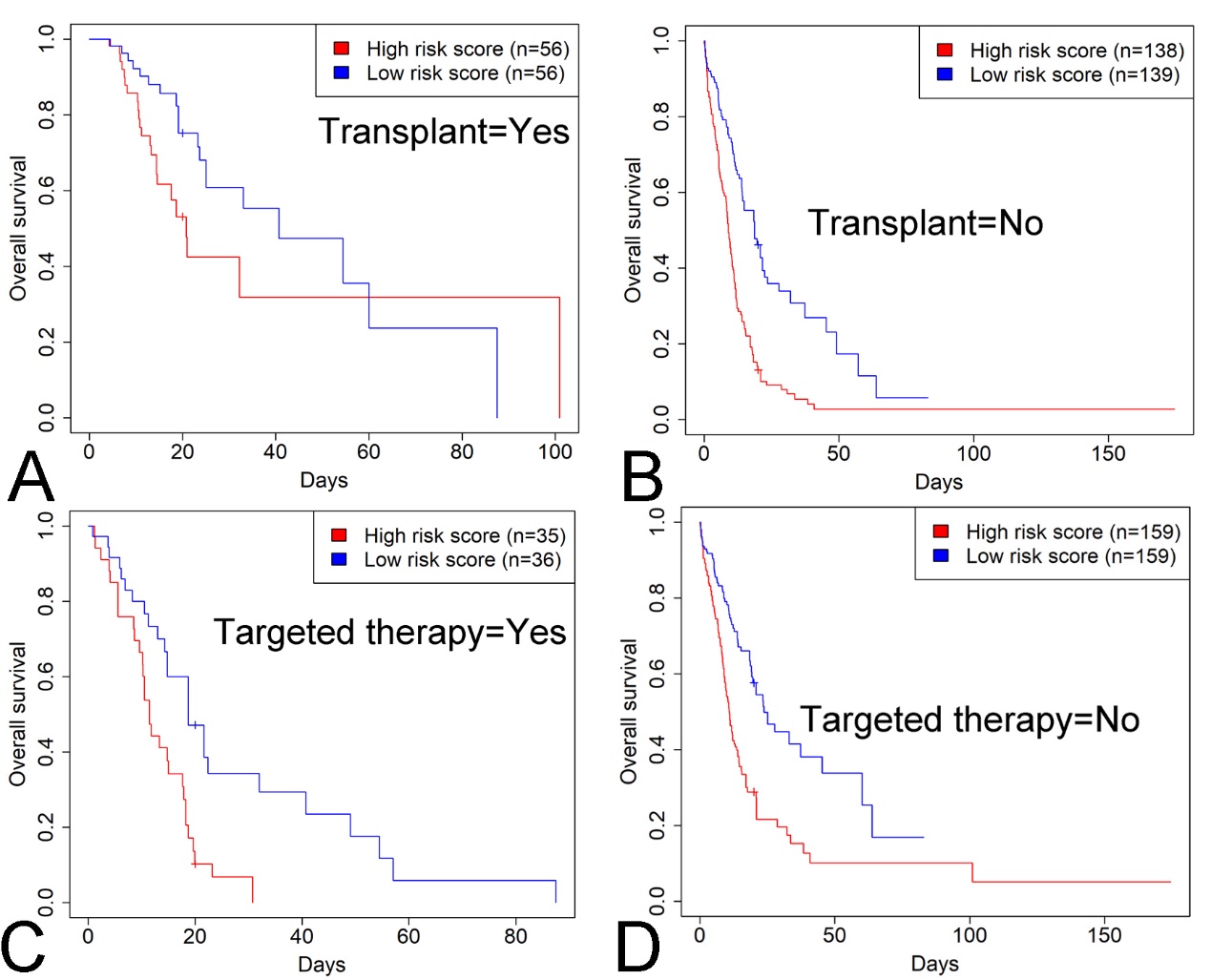
Supplementary Figure6. Kaplan-Meier survival analysis of patients’ OS with the risk score in the subgroups of LGG patients stratified by bone marrow transplant and targeted therapy (A-F) of the TCGA cohort.


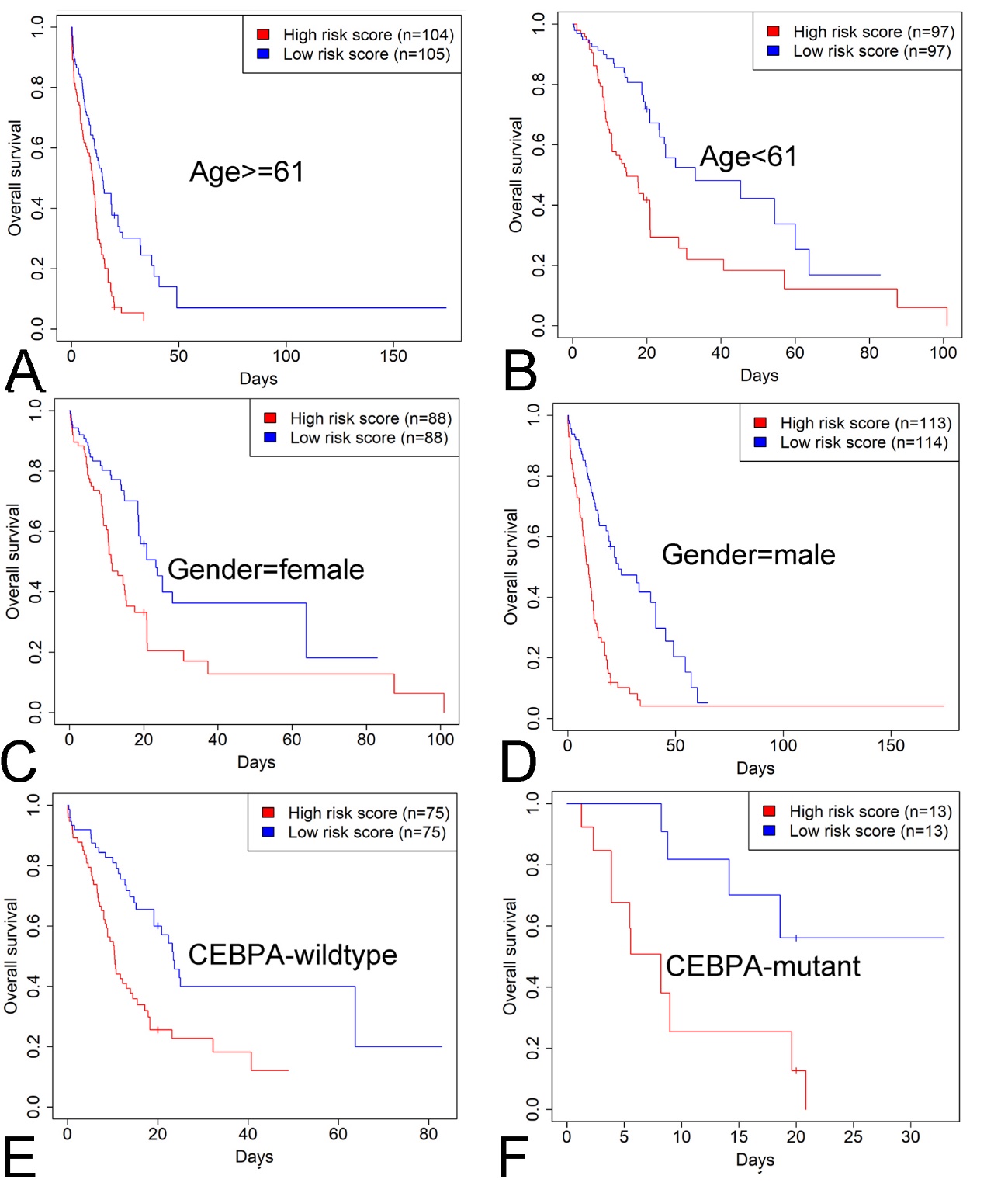
Supplementary Figure7. Kaplan-Meier survival analysis of patients’ OS with the risk score in the subgroups of LGG patients stratified by the median patient’s age, gender and *CEBPA* mutation (A-F) of the OHSU cohort.


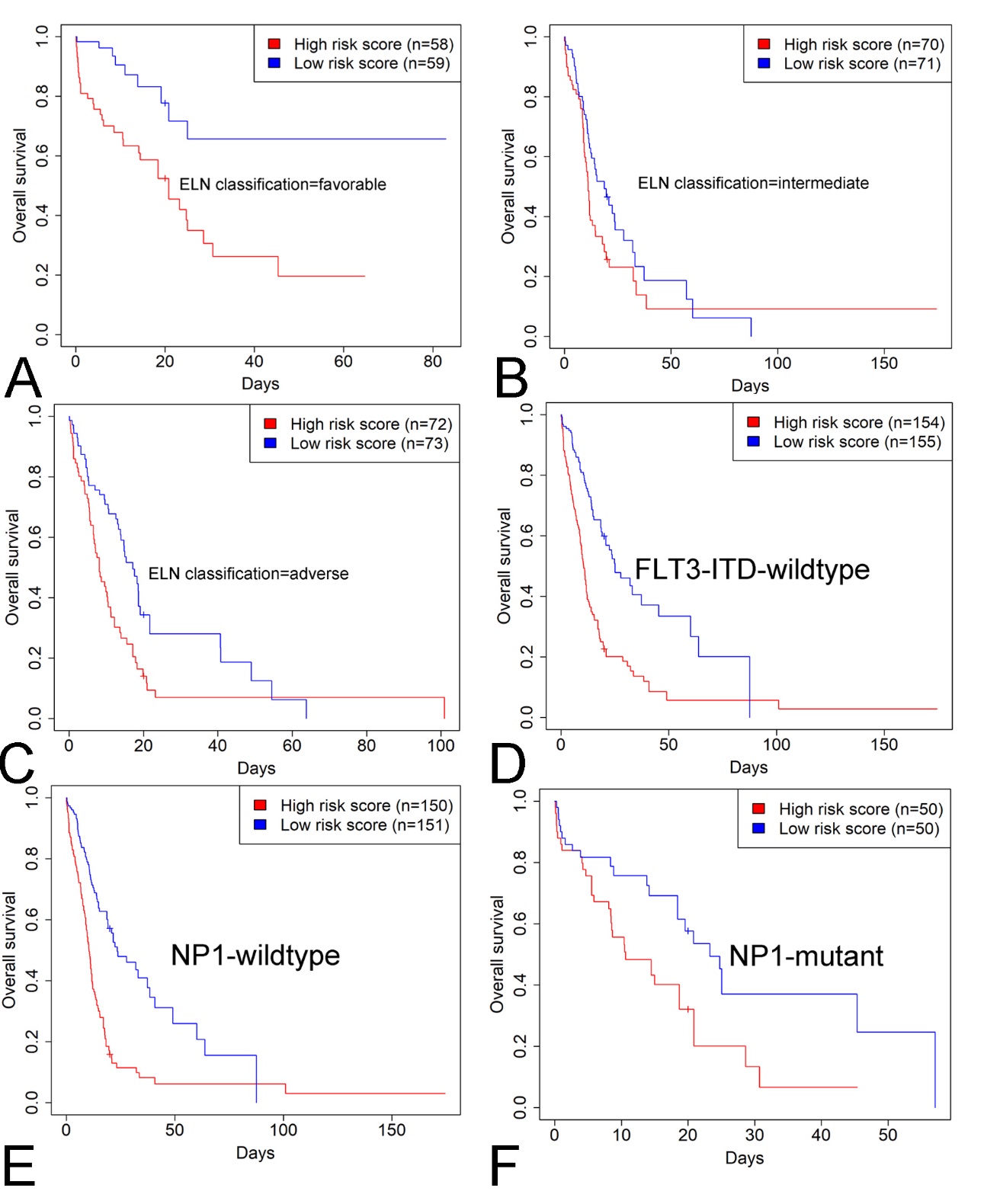
Supplementary Figure8. Kaplan-Meier survival analysis of patients’ OS with the risk score in the subgroups of LGG patients stratified by ELN classification, *FLT3-ITD* and *NP1* mutation (A-F) of the OHSU cohort.


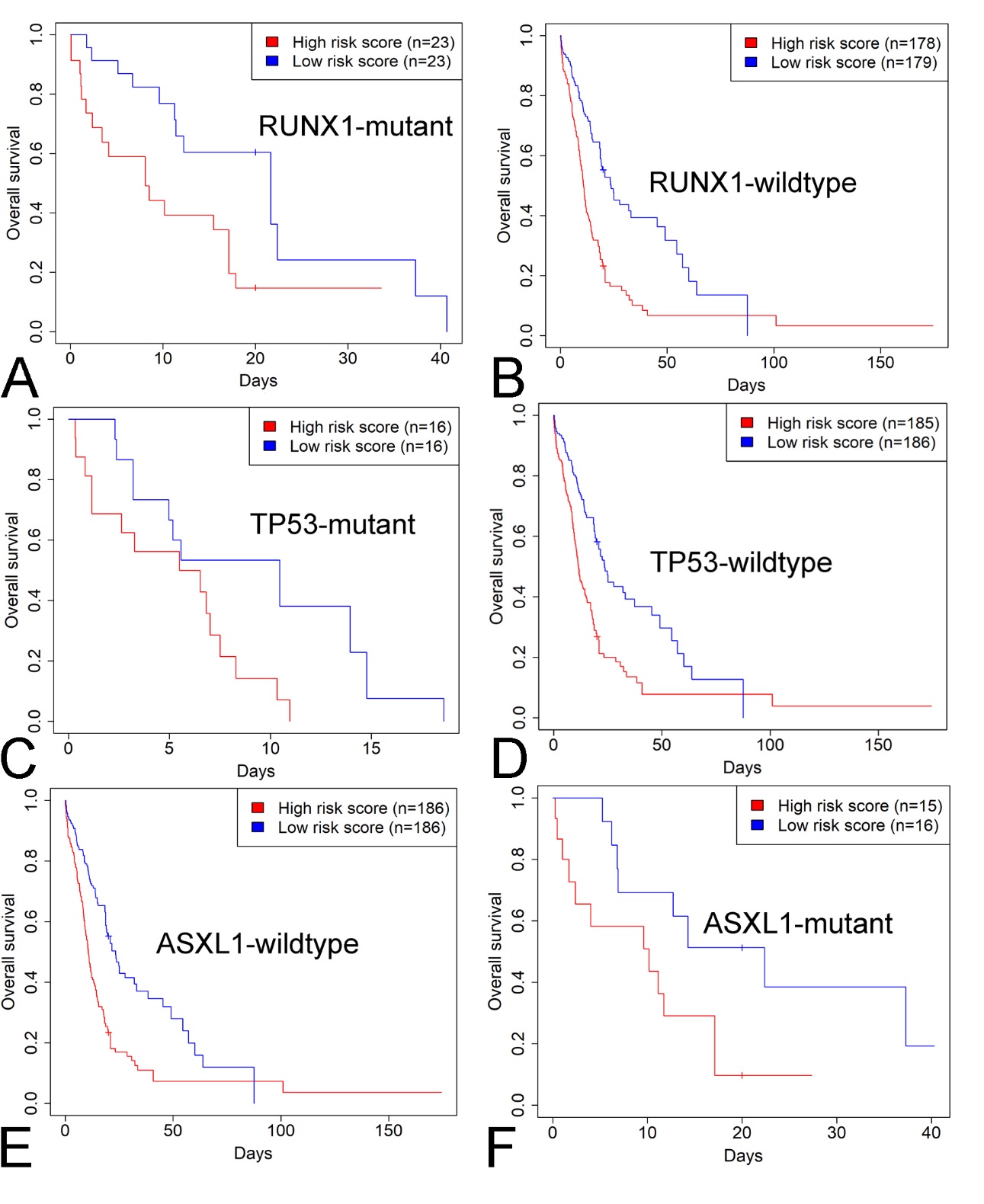
Supplementary Figure9. Kaplan-Meier survival analysis of patients’ RFS with the risk score in three subgroups of LGG patients stratified by *RUNX1*, *TP53* and *ASXL1* mutation (A-F) of the OHSU cohort.


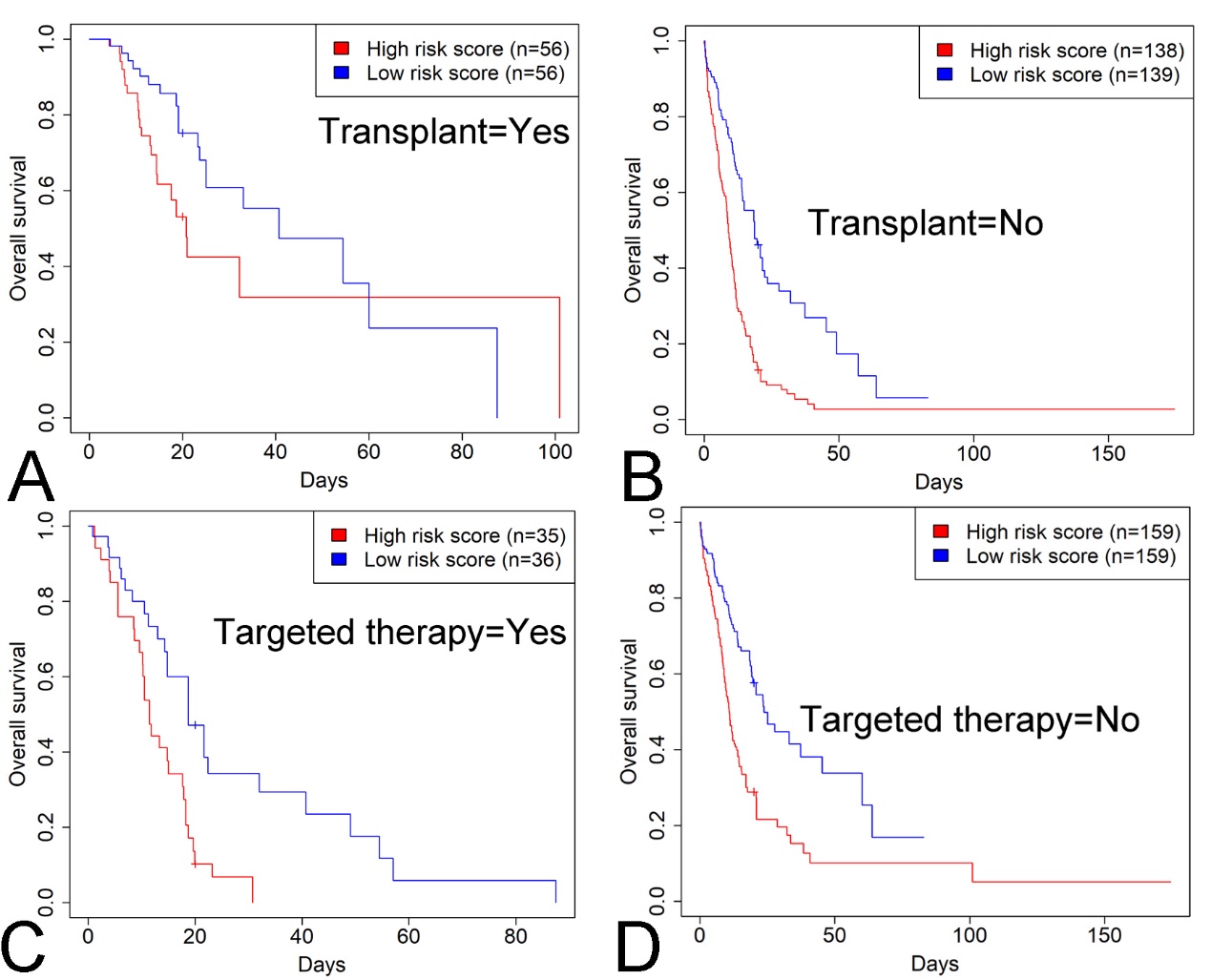


Supplementary Figure10. Kaplan-Meier survival analysis of patients’ RFS with the risk score in three subgroups of LGG patients stratified by bone marrow transplant and targeted therapy (A-D) of the OHSU cohort.
